# A cohort study investigating a simple, early assessment to predict upper extremity function after stroke - a part of the SALGOT study

**DOI:** 10.1186/s12883-015-0349-6

**Published:** 2015-06-18

**Authors:** Hanna C. Persson, Margit Alt Murphy, Anna Danielsson, Åsa Lundgren-Nilsson, Katharina S. Sunnerhagen

**Affiliations:** Department of Clinical Neuroscience and Rehabilitation, Institute of Neuroscience and Physiology, Sahlgrenska Academy, University of Gothenburg, Gothenburg, Sweden; Unit of Physiotherapy, Division of Health and Rehabilitation, Institute of Neuroscience and Physiology, Sahlgrenska Academy, University of Gothenburg, Gothenburg, Sweden

**Keywords:** Prognosis, Rehabilitation, Motor skills, Movement, Outcome assessment

## Abstract

**Background:**

For early prediction of upper extremity function, there is a need for short clinical measurements suitable for acute settings. Previous studies demonstrate correct prediction of function, but have ether included a complex assessment procedure or have an outcome that does not automatically correspond to motor function required to be useful in daily activity. The purpose of this study was to investigate whether a sub-set of items from the Action Research Arm Test (ARAT) at 3 days and 1 month post-stroke could predict the level of upper extremity motor function required for a drinking task at three later stages during the first year post-stroke.

**Methods:**

The level of motor function required for a drinking task was identified with the Fugl-Meyer Assessment for Upper Extremity (FMA-UE). A structured process was used to select ARAT items not requiring special equipment and to find a cut-off level of the items’ sum score. The early prognostic values of the selected items, aimed to determine the level of motor function required for a drinking task at 10 days and 1 and 12 months, were investigated in a cohort of 112 patients. The patients had a first time stroke and impaired upper extremity function at day 3 after stroke onset, were ≥18 years and received care in a stroke unit.

**Results:**

Two items, “Pour water from glass to glass” and “Place hand on top of head”, called ARAT-2, met the requirements to predict upper extremity motor function. ARAT-2 is a sum score (0-6) with a cut-off at 2 points, where >2 is considered an improvement. At the different time points, the sensitivity varied between 98 % and 100 %, specificity between 73 % and 94 %. Correctly classified patients varied between 81 % and 96 %.

**Conclusions:**

Using ARAT-2, 3 days post-stroke could predict the level of motor function (assessed with FMA-UE) required for a drinking task during the first year after a stroke. ARAT-2 demonstrates high predictive values, is easily performed and has the potential to be clinically feasible.

**Trail registration:**

ClinicalTrials.gov: NCT01115348

## Background

Each patient’s specific needs should be identified early after a stroke in order to reduce impairment, optimize rehabilitation and minimize costs. A common impairment after stroke is reduced upper extremity (UE) function which renders an increased risk of dependency on others and a prolonged in-patient stay [1]. Greater knowledge about the results of reduced UE after a stroke has been emphasized by patients as important [2], but the evaluation of UE functioning requires staff, equipment and time, which increases costs. Clinical scales, such as the Fugl-Meyer Assessment [3] and the Action Research Arm Test (ARAT) [4–6] are available for assessment of functioning in the UEs after stroke. Comprehensive clinical assessments can be time consuming or require special equipment, and might therefore be less suitable for screening in the acute setting.

Important factors predicting UE recovery after a stroke are initial motor function, stroke severity, and initial neuropsychological status [7–9]. Previous studies demonstrate that the presence of finger extension and shoulder abduction early post-stroke predicts UE functioning at six months [10–12]. A combination of the clinical assessment and transcranial magnetic stimulation (TMS) or magnetic resonance imaging (MRI) has also been shown to have high prediction accuracy at 12 weeks after stroke onset [13]. However, in these studies the assessment procedures are complex, or the outcome does not tell us if this predicted motor function can be useful for daily activities. A quickly administered clinical test predicting meaningful recovery of function, reflecting the patient’s ability to use their affected arm in a daily activity, would be warranted for efficient treatment planning.

The purpose of this study was to investigate whether a sub-set of items from ARAT, administered at 3 days and 1 month post-stroke, could predict the level of UE motor function (assessed with the FMA-UE) required for a drinking task, at 10 days and at 1 and 12 months after stroke onset.

## Methods

### Participants

Over a period of 18 months in 2009-2010, 117 patients from a stroke unit at the Sahlgrenska University Hospital in Gothenburg, Sweden, were consecutively included in the Stroke Arm Longitudinal Study at the University of Gothenburg (SALGOT study) [14], ClinicalTrials.gov: NCT01115348. The SALGOT study had the following inclusion criteria: 1) first clinical stroke (ischaemic or haemorrhagic), diagnosed according to the World Health Organization; 2) impaired UE function at day 3 (±1 day) after stroke onset, defined as <57 points on ARAT (0-57 points) [4, 6]; 3) receiving treatment in the stroke unit at 3 days after onset; 4) residency in the Gothenburg urban area; and 5) ≥18 years of age. The exclusion criteria were: 1) an UE injury/condition prior to the stroke that limited the functional use of the affected arm and hand; 2) severe multi-impairment or diminished physical condition before the stroke; 3) short life expectancy; and 4) non-Swedish speaking. In addition, in the present study, a 5^th^ exclusion criterion was used: 66 points on the Fugl-Meyer Assessment scale for Upper Extremity (FMA-UE) [3] (0-66 points) at 3 days post stroke. These criteria resulted in a cohort of 112 eligible patients. The SALGOT study received ethical approval from the Regional Ethical Review Board in Gothenburg. All patients, or their next of kin, provided written informed consent for participation.

### Clinical assessments and procedures

UE motor function at 3 days after stroke onset was assessed with the FMA-UE, [3, 15] consisting of 33 items, each scored 0-2 and summed to a total score of 0-66 points. The total score on the FMA-UE was stratified into two groups: severe UE impairment (≤31) and mild/moderate UE impairment (≥32). A score of FMA-UE ≥32 was used to identify patients from the SALGOT cohort who possessed the motor function required for a drinking task with the paretic arm and used as the cut-off between the two groups. This cut-off was based on a previous study [16], in which patients were included if they were able to perform a drinking task with their affected arm (lowest score for FMA-UE was 32 points). This drinking task requires the capacity to reach, grasp, lift, transport the glass as well as drink. Validation of the cut-off score of FMA-UE ≥32 to correctly classify patients’ motor ability to drink from a glass (the drinking task) was based on the entire cohort. The results were: at 10 days sensitivity 98 % (CI 95 % 0.91-1.0) and specificity 89 % (0.77-0.96), at 1 month sensitivity 100 % (0.92-1.0) and specificity 93 % (0.84-0.98) and at 12 months sensitivity 100 % (0.85-1.0) and specificity 96 % (0.87-1.0). These results confirmed the use of this cut-off in the subsequent analysis. The majority of classification errors occurred in data gathered at 10 days post stroke, on the group of patients with moderate/mild UE impairment but with a poor hand function and inability to grip and perform the drinking task (n = 6).

**Fig. 1:**
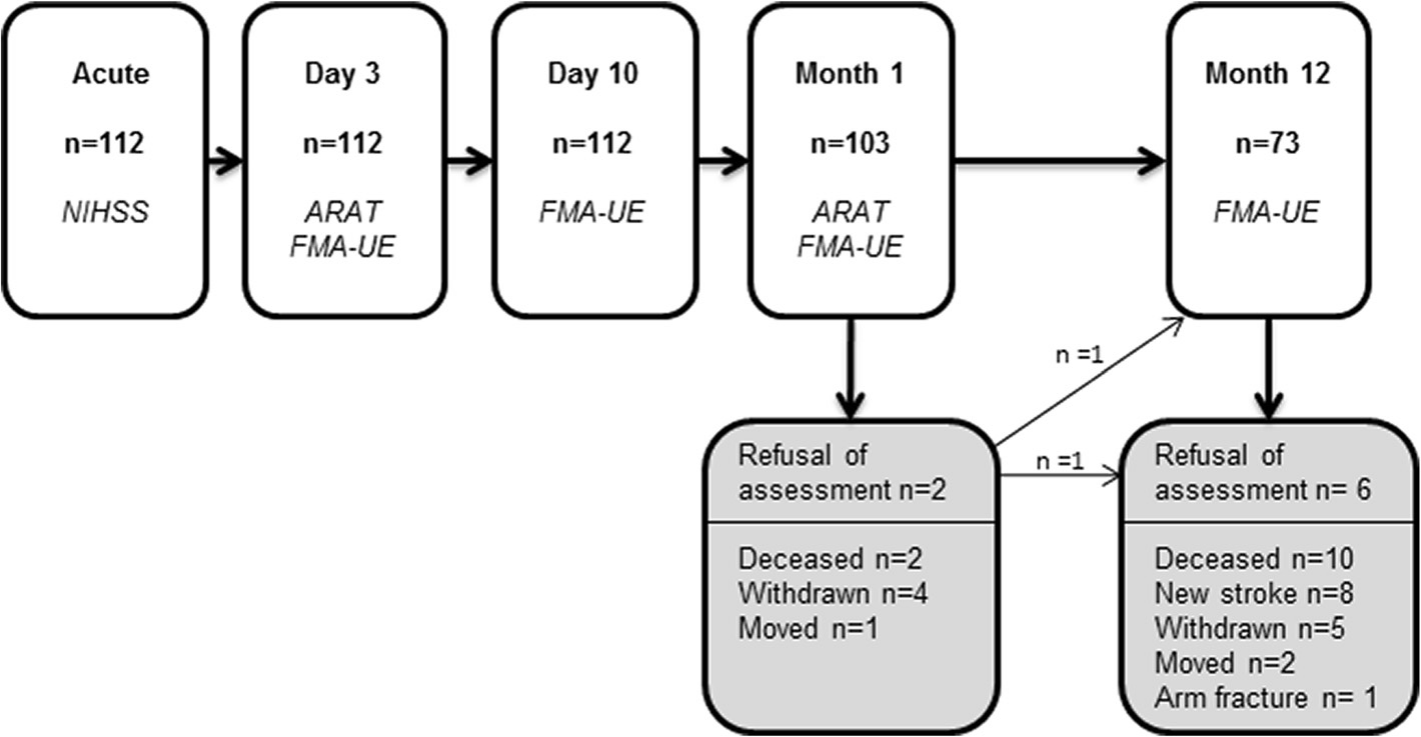
Flowchart of the assessments and the drop-outs at different time points

Three physiotherapists, after joint training, performed the clinical assessments according to a standardized procedure [14]. The majority of the assessments were performed at the hospital, and if the patient was unable to travel, assessment was conducted in the patient’s home, nursing home or rehabilitations unit. Global neurological deficits were detected by physicians using the National Institute of Health Stroke Scale, NIHSS (0 = no deficit, 42 = severe neurologic deficit) at arrival [17]. Type of ischemic stroke was classified according to the Bamford classification [18]. The assessment procedure is shown in Fig. 1. The patients received individually adjusted, functional task-specific rehabilitation from the first day at the stroke unit. Physiotherapist and occupational therapist were available in the primary care system as well as in the community and in nursing homes. The level of rehabilitation received at the different test occasions during the first year is described in Table 1, and follows the Swedish national guidelines [19].

**Table 1:** Characteristics at day 3 post-stroke, presented for the total group of patients and subgroups

	Total	FMA-UE ≤31 points	FMA-UE ≥32 points
Number of patients, n (%)	112 (100)	62 (55)	50 (45)
Age, years, mean (SD)	69 (13.0)	71 (12.3)	67 (13.6)
Men, %	55	53	58
Independence in mobility pre-stroke, %	94	92	96
Location of stroke, %			
*Right*	52	56	48
*Left*	42	42	42
*Bilateral*	4	0	2
*Brain stem*	1	0	1
*Unknown*	1	2	0
Ischaemic stroke, %	83	79	88
Intracerebral haemorrhage, %	17	21	12
Type of ischaemic stroke, Bamford, %			
*Total anterior cerebral infarct*	16	27	5
*Lacunar anterior cerebral infarct*	34	16	55
*Partial anterior cerebral infarct*	43	53	35
*Posterior circulation infarct*	7	4	9
Treated with thrombolysis, %	12	15	8
Treated with thrombectomy, %	4	5	2
Stroke Severity, NIHSS (0-42 p)			
*median, (q1-q3)*	7 (3-13)	12 (8-17)	4 (3-6)
Dominant arm affected, %	45	43	46
UE functioning day 3, FMA-UE (0-66 p)			
*median, (q1-q3)*	18 (4-55)	4 (2-10)	56 (47-61)
UE functioning day 3, ARAT (0-57 p) *median, (q1-q3)*	4.5 (0-43)	0 (0-3)	46 (35-51)
Obtained rehab^a^, %			
*Day 3, inpatient rehab, n = 112*	100	100	100
*Day 10, rehab/no rehab, n = 112*	93/ 7	98/2	82/18
*1 Month, rehab/no rehab, n = 104*	82/ 18	97/ 3	63/37
*12 Month, rehab/no rehab, n = 74*	38/62	59/41	16/84

^a^Obtained PT and/or OT intervention

Abbreviation: *ARAT*, The Action Research Arm Test; *Bamford* classification; *FMA-UE*, the Fugl-Meyer Assessment Scale for Upper Extremity; *NIHSS,* National Institutes of Health Stroke Scale; *OT*, Occupational therapist; *Rehab,* Rehabilitation; *PT*, Physiotherapist; *q1-q3* 1^st^ and 3^rd^ quartile values; *UE*, upper extremity

### ARAT and reduction of items

**Fig. 2:**
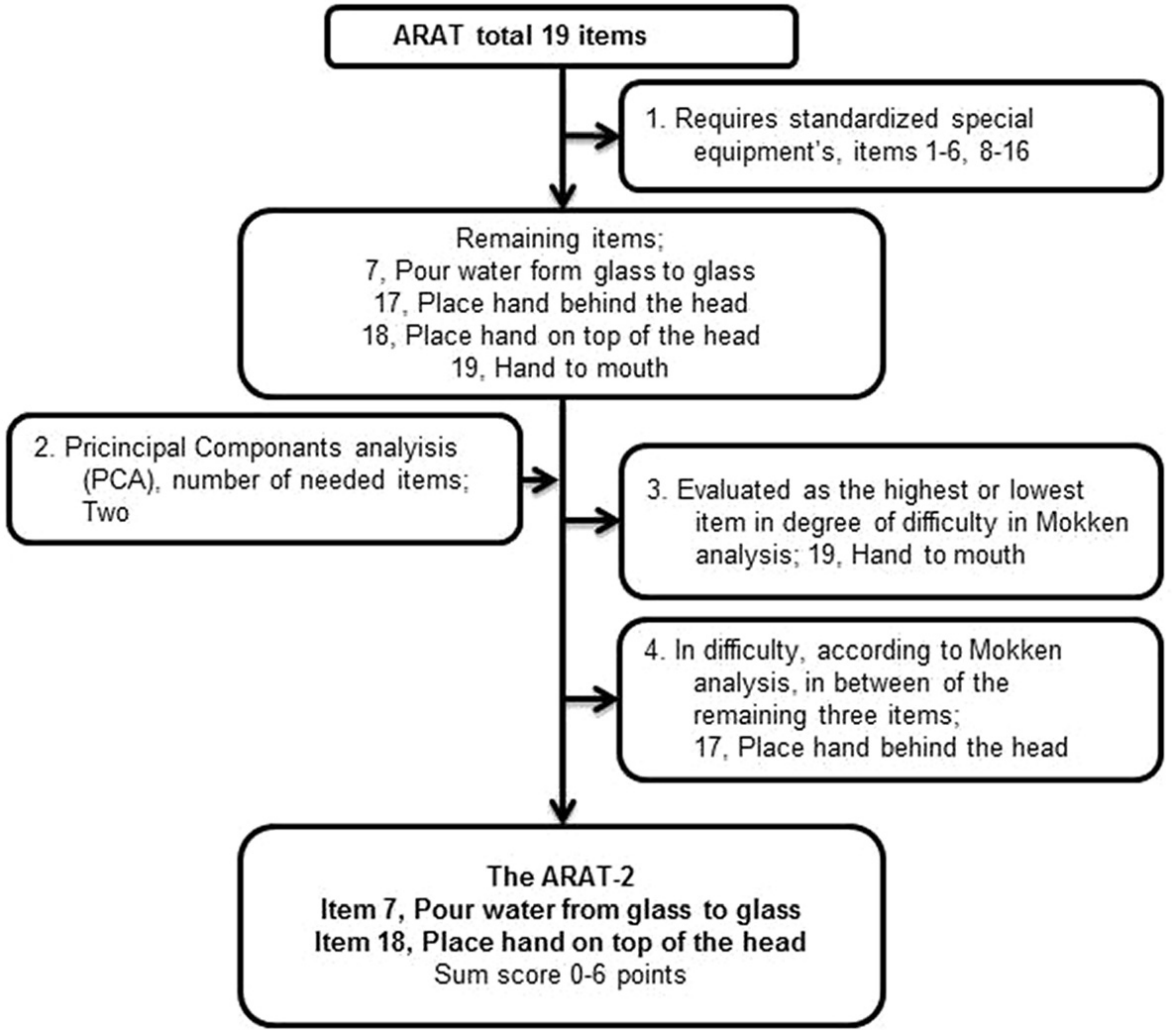
Illustration of the selection process to identify items from the Action Research Arm Test (ARAT) feasible for clinical use in the acute stage to predict the motor function required for a drinking task (FMA-UE ≥32 points), within the first year after stroke

The ARAT [4–6] consists of 19 items, and the performance of each item is scored on a 4-point ordinal scale ranging from 0 (no task performance) to 3 (normal task performance) and summed to a total score of 0-57. The ARAT was performed in a standardized manner [6, 20] at 3 days and 1 month post-stroke. These time-points were considered as being of possible clinical importance for both early and long term rehabilitation planning. The procedure for choosing a sub-set of items from the ARAT was conducted in four steps (Fig. 2): 1) items not requiring special equipment were identified (consensus among the authors and physiotherapists at the stroke unit); 2) the minimum number of items needed to capture most of the variance in the ARAT at day 3 was explored; 3) according to published results of a Mokken scale analysis [21], items identified by their means as highest or lowest in degree of difficulty were excluded; and 4) from the same analysis [21] the two remaining items with the greatest distance in their means were selected, in order to identify UE function at various degrees of stroke severity. From the selected sub-set of ARAT items, a cut-off level with potential to be clinically useful was determined where a higher score indicates better function.

The selected ARAT items from 3 days post stroke were used to predict the UE function (to detect the motor function required for a drinking task using the paretic arm) at 10 days, 1 month and 12 months post stroke; and the ARAT items from 1 month post stroke were used to predict the UE function at 12 months post stroke.

### Data handling and statistics

To enable the use of data from 18 patients not assessed at day 10 using the FMA-UE (due to administrative problems), an estimated score for each patient was obtained in the following manner: The mean change from day 3 to day 10 for all patients assessed at both time points (n = 94) was calculated and added to each of the 18 patients’ day 3 FMA-UE scores. An estimated value at day 10 could not exceed the score of the subsequent assessment.

Age differences between the groups were analysed with an independent *t*-test, categorical variables with Mann–Whitney *U* test and dichotomized variables with Fisher’s exact test. Correlations between all of the ARAT variables were investigated with Spearman’s rank correlation (rho). The fewest number of items needed was verified using principal components analysis (PCA) based on the total population (n = 117). Only components with eigenvalues ≥1 were selected and items with loading values greater than 0.6 were considered, according to Kaiser’s criterion [22]. Using receiver operation characteristic (ROC) curves, an optimal cut-off level of the score of the sub-set of ARAT items for all time points was identified. The maximum sensitivity and specificity levels were estimated with preference given to the sensitivity. The score of the sub-set of ARAT items was analyzed with 2-way contingency tables at day 10, month 1 and month 12. The sensitivity, specificity, positive predicted value (PPV), negative predicted value (NPV), percentage of correctly classified patients, and likelihood ratios were calculated at each time point, including 95 % exact confidence intervals (CIs) [23, 24]. To provide strong evidence in most circumstances (in a stroke population) positive likelihood ratio values should be above 10 and negative likelihood ratios should be below 0.1 [25]. The statistical analysis was conducted using IBM Statistical Package for Social Sciences (SPSS version 21.0) for Windows, and the CI of likelihood ratios were calculated using the Prop CIs Package in R version 3.1.1 (2014-07-10).

## Results

In total, all 112 patients were assessed at days 3 and 10, 9 patients (8 %) were lost to follow-up at 1 month, and 39 patients (35 %) were lost to follow-up at 12 months (Fig. 1). There were no significant differences between the sex of the participants, initial stroke severity or initial UE function when comparing patients who completed the study (n = 73) and those who dropped out at 12 months (n = 39); however, the drop-outs were, on average, 6.6 years older, (p = 0.017). The clinical characteristics of the 112 patients are presented for the whole group and for the two subgroups (FMA-UE cut off ≥32 points) in Table 1.

**Fig. 3:**
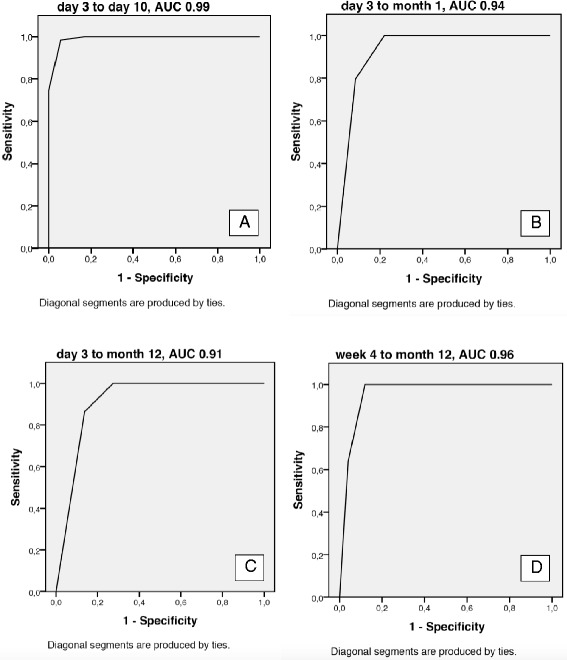
Illustration of the properties of the two item “Pour water from glass to glass” and “Place hand on top of head” (sum score 0-6 points) from the Action Research Arm Test (ARAT), to predict the patient’s ability to have the motor function required to use the paretic arm in a drinking task (FMA-UE ≥32 points), using receiver operating characteristic (ROC) curves. The optimal cut-off level at 2 points in the different assessments (A-D) is presented. **A)** the assessment at day 3 predicting day 10, **B)** the assessment at day 3 predicting month 1, **C)** the assessment at day 3 predicting month 12, **D)** the assessment at month 1 predicting month 12. Abbreviation: *ACU*, area under the curve

The correlations between the ARAT items varied between rho 0.736 and 0.981. The PCA of the ARAT identified one factor (dimension) with two components with eigenvalues ≥1, which together explained 95.1 % of the total variance. Furthermore, the PCA showed that all of the items had a component value >0.875, indicating that any of the items could be used in a sub-set. Following the criteria of the selection process as shown in Fig. 2, the final two remaining items were “Pour water from glass to glass” and “Place hand on top of head”, which comprised the ARAT-2. The ARAT-2 score was composed as the sum score of the two original items (0-6). Figure 3 shows the predictive value of the ARAT-2 to classify the motor function required for a drinking task (FMA-UE ≥32 points) and a cut-off level of 2 points on the ARAT-2 was identified as appropriate to predict outcomes at day 10, month 1 and month 12. The greatest area under the curve was observed from day 3 to day 10: 0.99 (Fig. 3, a).

Correctly classified patients, identified by 2-way contingency tables using the ARAT-2 from day 3 to day 10, was 96 % (CI 95 % 0.91-0.99), at 1 month 87 %, (CI 95 % 0.80-0.93), and at 12 months 81 % (CI 95 % 0.70-0.89). Using the ARAT-2 from 1 month, correctly classified patients at 12 months was 92 % (CI 95 % 0.83-0.97). Table 2 shows the sensitivity, specificity, and predictive values of the ARAT-2 for each time point. In summary, the predictive values of the ARAT-2 to correctly classify patents level of UE motor function required for a drinking task were shown to be highest from day 3 to day 10. Furthermore, the ARAT-2 had high sensitivity and NPV and negative likelihood ratios at every time point, indicating a high probability to accurately predict motor function required for the drinking task in patients who initially had >2 points on ARAT-2. Lower specificity and PPV and positive likelihood ratios were observed from day 3 to 1 month and from day 3 to 12 months.

**Table 2:** Sensitivity, specificity, predictive values, and likelihood ratios for ARAT-2 at the different assessments

	Day 3-10 days *(CI)*	Day 3-1 Month *(CI)*	Day 3-12 Months *(CI)*	1-12 Months *(CI)*
Sensitivity	0.98 *(0.91-1.0)*	1.00 *(0.92-1.0)*	1.00 *(0.85-1.0)*	1.00 *(0.85-1.0)*
Specificity	0.94 *(0.84-0.99)*	0.78 *(0.64-0.87)*	0.73 *(0.58-0.84)*	0.88 *(0.76-0.96)*
PPV	0.95 *(0.86-0.99)*	0.77 *(0.65-0.88)*	0.61 *(0.44-0.77)*	0.79 *(0.59-0.92)*
NPV	0.98 *(0.90-1.0)*	1.00 *(0.92-1.0)*	1.00 *(0.91-1.0)*	1.00 *(0.92-1.0)*
LR^+^	17.36 *(6.40-50.60)*	4.54 *(2.93-7.49)*	3.64 *(2.44-5.84)*	8.33 *(4.20-17.80)*
LR^−^	0.02 *(0.00-0.096)*	0.00 *(0.00-0.10)*	0.00 *(0.00-0.21)*	0.00 *(0.00-0.17)*

Abbreviations: *CI*, 95 % exact confidence interval; *LR* ^*+*^, the positive likelihood ratio; *LR* ^*−*^, the negative likelihood ratio; *NPP*, negative predictive value; *PPV*, positive predictive value

## Discussion

Information from a sub-set of two items from ARAT used at 3 days post-stroke showed a high ability to correctly predict the level of motor function needed for a common daily task at 10 days, 1 and 12 months after stroke. The items, structurally selected to be clinically feasible, were “Pour water from glass to glass” and “Place hand on top of the head”, named ARAT-2. Based on assessment at 3 days post-stroke the ARAT-2 was shown to have very high NPVs (95-100 %) at 10 days, 1 and 12 months, indicating accurate prediction of patients having some arm and hand function (ARAT-2 > 2) at an early stage. The PPVs during the first year indicated less accurate prediction in patients with no or very little arm and hand function (ARAT-2 ≤ 1) at day 3. The difficulty to correctly predict UE motor function in patients with initially low function has previously been shown [13, 26]. One possible reason for this limitation may be the stroke severity itself and that restitution of motor function, as well as rehabilitation with sufficient intensity starts later for these patients [27]. Another explanation could be that the clinical assessment scores at stroke onset does not give enough information to distinguish between patients with similar initial impairment but with different recovery potential and thereby predicted outcomes [13].

To perform the items in ARAT-2*,* some initial function in shoulder abduction, flexion, elevation and finger extension is required. The item in ARAT, “Pour water from glass to glass”, consists of a similar movement as when drinking, but to receive one point the patient only needs to initiate the task. Initial function in shoulder abduction and finger extension has been described as important predictors for outcomes in UE function at 6 months [10, 12]. One of these studies [10] based on patients with anterior circulation stroke, used a cut-off level ≤10 on the ARAT which does not automatically reflect recovery of function or ability meaningful for use of the arm in an activity [28]. It is not clear if that cut-off provides sufficient information to guide clinical decisions about the predicted ability to use an upper extremity in an activity [29]. When combining the clinical assessment with TMS or MRI using an algorithm [13], the accurate prediction of motor function increased, particularly in patients with low function initially, but this would also increase the cost and would not be feasible in all stroke units or hospitals. A complex algorithm could also be a barrier to applying prognostic models in routine care [30]. A recently published review [31] on prediction of motor recovery summarizes the overall prediction accuracy of outcome in 3 different recovery levels on ARAT at 6 months post stroke and compares 3 different approaches of correct prediction. Two gave less correct prediction compared to the approach in the present study; one with a competent prediction of experienced therapists and another using clinical assessment (finger extension and shoulder abduction), assessed within 72 h or at discharge. The third approach with a combination of clinical assessment and TMS or MRI evaluation, using an algorithm [13] yielded sufficient information to predict 82 % accurately. In the present study using ARAT-2, very high overall correct classification ability (81 %) from day 3 to 12 months was shown using only two items. The new ARAT-2 does not cover all aspects of functioning, but it could be considered useful as an early predictor of UE motor function that is required for a daily activity, such as drinking from a glass after a stroke. The ARAT-2 requires no special equipment, is quick and easy to use and has potential to contribute with valuable predictive and clinically useful information.

The original ARAT has good measurement properties [5, 6, 20, 32, 33] and has been frequently used in research, as well as in clinical practice, in many countries. To administer the total ARAT a special test kit is required and the test is rather time-consuming. To be clinically feasible in an acute setting, a predictive test should be easy to administer, include few items, not require any special equipment and be useful both in severe and moderate/mild impairments.

In the present study the PCA showed that only two components were needed from the ARAT to capture most of the variance and it was desirable that two items could predict the function of patients with mild stroke, as well as severe stroke. The items’ differences in difficulty were identified with a Mokken analysis [21] in which similar differences in difficulty also were shown using Rasch analysis. This provided further support to our theory of the items’ ability to detect patients with different severities of motor function impairment.

This study was aimed to be particularly relevant to clinical practice. Sufficient motor function required for a daily activity was chosen as an outcome and the different time points selected were relevant for rehabilitation planning and outcome assessment. This information can be used for planning of the content of rehabilitation; i.e. training vs compensatory strategies as well need for care and assistance. To be able to use the upper extremity in a drinking task is essential for daily activities, and could therefore be seen as an important clinical goal, which is also stated in a consensus document published by stroke survivors, caregivers and health professionals [2]. The task includes both reaching and grasping movements which are essential for upper extremity use, in a functional activity. In this study we tried to cover both, the need for early evaluation (day 3 and day 10) since the length of stay in hospital is getting shorter, as well as the need for a later assessment, at 1 month, which can be useful for more impaired patients who will require a longer period of rehabilitation [34].

The likelihood ratios at day 3 to day 10 were high, indicating the possibility of using the ARAT-2 as a screening test, to predict the level of motor function required for a drinking task. However, at the later time points, the likelihood ratios, including broad confidence intervals, indicated that it could be problematic to use the ARAT-2 for late screening. The positive likelihood ratio increased when using the assessment undertaken at 1 month (compared to the assessment results from day 3) for prediction at 12 months, but not sufficiently to indicate high accuracy in the general stroke population [25].

Some limitations of this study should be noted. Firstly, the dichotomization of the FMA-UE score reduced the overall information from the data. Using a cut-off of the FMA-UE (≥32 points) was, with high sensitivity and specificity, shown to correspond to the ability to use the arm to perform a drinking task. Secondly, to calculate the predictive properties of the ARAT-2, 2-way contingency tables were used due to the limited number of patients in some of the categories (100 % correct prediction in some categories). A 2-way contingency table limits the possibility to adjust for confounding variables such as age, sex or sensory function. Also the fact that the ARAT was not used to directly predict the drinking task is a limitation. Moreover, the present study had a drop-out of 35 % at the final assessment after 12 months, mainly due to deaths, new strokes or withdrawal from the study. Other potential reasons for the remaining drop-outs could be the time point for inclusion close to stroke onset, when consequences of the stroke still were unclear for the patient; this might have led to the inclusion of patients who only later realized their limited ability to participate in the study. Another reason could be the broad inclusion criteria, which were intended to capture a total population in a stroke unit with impaired UEs, facilitating inclusion of different levels of stroke severity, UE impairment and cognitive function. This might have led to a higher drop-out rate, but in contrast, an un-selected population after stroke with impaired UEs was achieved.

In future studies, the discriminative validity using a cut-off at FMA-UE score of ≥32 to identify persons that are able to perform a drinking task needs to be assessed in another stroke cohort. Similarly, the predictive validity of ARAT-2 to directly predict a drinking task needs to be investigated in another independent cohort.

## Conclusions

The ARAT-2 requires no special equipment, is feasible in the acute setting, and provides information on the expected UE function required for a drinking task during the first year post-stroke. The ARAT-2 has a potential to be implemented in the acute setting and in the stroke unit, and it could contribute to the knowledge of a patient’s probable UE function at later stages.
